# Lymphopenia and elevated NLR identify patients with septic shock who may benefit from corticosteroids: a target trial emulation study

**DOI:** 10.1186/s40635-026-00972-8

**Published:** 2026-09-29

**Authors:** Patrick Jason Toppin, Mario Diego Teles Correia, Yale Chang, Lasith Adhikari, Brian McLaverty, Annamalai Natarajan

**Affiliations:** 1 Section of Anaesthesia and Critical Care, University of the West Indies, Mona Kingston, Jamaica; 2 Real Hospital Portugues, Intensive Care Department, Avenida Agamenon Magalhães (Avenue), Paissandu Recife, 4760 Pernambuco Brazil; 3 Intensive Care Department, IMIP, Instituto de Medicina Integral, Rua dos Coelhos, 300 , Boa Vista Recife, Pernambuco Brazil; 4 Hospital Patient Monitoring, Philips North America, 222, Jacobs Street, Cambridge, 02141 MA USA; 5 2US FCRM Modelling and Advanced Analytics, TD Bank Group, 200 State Street, Boston, 02109 MA USA

**Keywords:** Septic shock, Corticosteroids, Neutrophil-to-lymphocyte ratio, Platelet-to-lymphocyte ratio, Precision medicine, Target trial emulation

## Abstract

**Background:**

Septic shock is the most severe form of sepsis and has high mortality. Despite the widespread use of corticosteroids, evidence of their benefit remains unclear; precision critical care medicine has sought to identify which patients will benefit. The neutrophil-to-lymphocyte ratio (NLR) and platelet-to-lymphocyte ratio (PLR) are widely available markers of inflammation and immune function. This study aimed to determine whether these parameters can identify septic shock patients likely to benefit from corticosteroid treatment.

**Methods:**

A target trial was emulated using data from the Medical Information Mart for Intensive Care IV database. Adult patients in the ICU for at least 24 h who met the Sepsis-3 definition for septic shock were included. The cohort was divided into those who received corticosteroids and those who did not. Covariates extracted included NLR and PLR. An Augmented Inverse Probability Weighting (AIPW) double-robust estimator was applied to evaluate the effect of corticosteroids on ICU mortality (primary outcome), in-hospital mortality, the need for renal replacement therapy, the duration of vasopressor therapy, and the length of ICU and hospital stay (secondary outcomes). Univariate AUROC was used to compare the predictive performance of the evaluated parameters.

**Results:**

5,533 septic shock stays were identified; 1,009 (18.23%) received corticosteroids. The corticosteroid group was much sicker than the control group, with higher Sequential Organ Failure Assessment (SOFA) scores and greater vasopressor requirements. After adjusting for confounding via an AIPW doubly robust estimator, the analysis revealed that lower baseline lymphocyte count (AUROC 0.65), higher NLR (AUROC 0.62), and higher PLR (AUROC 0.63) had modest but consistent ability to identify cases that benefited from corticosteroid therapy, as reflected by reduced ICU mortality. These compared favourably to the use of the maximum SOFA score (AUROC 0.65) and had similar predictive power for all secondary outcomes.

**Conclusion:**

Lower lymphocyte count, higher NLR and PLR were associated with an increased likelihood of model-estimated benefit from corticosteroids. Although the predictive power of these markers was modest, their low cost and wide availability could make them appealing in low-resource settings. Further research into these simple parameters to guide the treatment of septic shock is warranted.

**Supplementary Information:**

The online version contains supplementary material available at https://doi.org/10.1186/s40635-026-00972-8.

## Background

Sepsis is an unregulated immune response to infection, resulting in organ dysfunction [1]. Septic shock is sepsis with associated circulatory failure and systemic hypoperfusion, resulting in markedly increased mortality and cost [2–5]. In 2017, there were an estimated 48.9 million cases of sepsis and 11 million deaths, accounting for almost one-fifth of all deaths globally [2]. The literature on the use of corticosteroids in septic shock is extensive and discordant. Trials before the year 2000 used high doses of corticosteroids and reported increased mortality and complications [6, 7]. The highly influential study by Annane found that a combination of a low-dose corticosteroid and a mineralocorticoid receptor antagonist reduced mortality in patients with septic shock [8]. Following this study, hydrocortisone was widely used to manage septic shock. However, several large, well-conducted trials failed to reproduce this reduction in mortality, despite evidence of improved blood pressure [9–12].

Some have suggested that the discordance in corticosteroid response is due to heterogeneity among patients with septic shock [13–16] and have highlighted the need to identify patients likely to benefit from corticosteroids. This discordance is highlighted by the results of two major trials, the CAPE-COD and REMAP-CAP trials [17, 18]. The CAPE-COD trial examined the effect of hydrocortisone on patients with severe community-acquired pneumonia but excluded those with septic shock [17]. It concluded that hydrocortisone resulted in a reduction of mortality. The REMAP-CAP examined a similar patient population but included patients with shock [18]. It concluded that there was unlikely to be a significant benefit from corticosteroid use in the patient population. The discordance in results has reawakened interest in identifying which patients will benefit from corticosteroids. Strategies used to predict response to corticosteroids have included evaluation of adrenal function [8, 19] and measurement of markers of inflammation and immune function, from cytokine levels to specific genes and transcription of specific proteins [20–23]. None of these strategies is currently widely used, either due to poor discriminatory ability or limited availability due to cost.

Haematological parameters such as the neutrophil-to-lymphocyte (NLR) and platelet-to-lymphocyte (PLR) ratios are easily computed from readily available blood tests. The NLR and PLR assess the balance between the innate immune response and inflammation, modulated by neutrophils and associated with platelet count, and the more specific acquired immune response, regulated by lymphocytes [24–26]. In a retrospective review of over 1200 patients with sepsis from the MIMIC-IV database, the NLR and neutrophil-to-platelet ratio were found to have superior predictive power for 28-day mortality compared with the SOFA score (AUCs of 0.66 and 0.56, respectively) [27]. A meta-analysis of nine studies with over 10,000 patients confirms the prognostic value of the NLR in patients with sepsis, as higher values were associated with increased 28-day mortality [28]. Increased PLR values have similarly been associated with increased mortality in patients with sepsis [27].

Given the central roles of inflammation and immune dysfunction in the pathophysiology of sepsis and septic shock, parameters such as the NLR and PLR are appealing ways to assess inflammation and the immune response [26], identify clinical subtypes, and possibly guide therapy. The case for using the NLR is well presented in a letter by Lafon and colleagues, which reviews the correlation between the widely available NLR and the more expensive monocyte HLA-DR (mHLA-DR) levels in septic patients [29]. They suggest it may be a simple method to monitor immune dysfunction during sepsis and guide therapy.

In a target trial emulation study, Rajendran et al. used machine learning techniques to identify subtypes of septic patients and patients who may benefit from corticosteroids [30]. They noted that patients with worsening organ function, as measured by SOFA score, and a reduced adaptive immune response were more likely to benefit from corticosteroids. Wittermans et al. evaluated the ability of NLR, neutrophil, and lymphocyte counts to identify patients with community-acquired pneumonia who would benefit from dexamethasone [31]. The study found that patients with higher NLR, total white cell count, and neutrophil count had a shorter length of hospital stay when given dexamethasone. In a more recent study, Bologheanu et al. used reinforcement learning to predict clinical response to steroids in patients with sepsis [32]. They found that leucocyte count was one of the most influential parameters in their model. These studies support the potential of variations in these haematological parameters to identify patient subtypes with septic shock and to predict the response to corticosteroids.

To the best of our knowledge, no studies have examined the role of these widely available haematological parameters in identifying septic shock subpopulations that respond to corticosteroid treatment. This can support the development of precision medical techniques that are available in low-resource settings.

## Methods

### Aims/objectives

The aim of this study was to evaluate if the NLR, PLR, neutrophil, and lymphocyte count at the onset of septic shock can predict which patients will benefit from corticosteroid therapy. The primary outcome used to determine benefit from corticosteroid therapy was ICU mortality. Secondary outcomes were in-hospital mortality, ICU length of stay (LOS), Hospital LOS, the need for renal replacement therapy (RRT), and the duration of vasopressor administration.

### Dataset

This study utilised data from patients admitted to the ICU at Beth Israel Deaconess Medical Centre (BIDMC), extracted from the Medical Information Mart for Intensive Care (MIMIC-IV) database. The MIMIC-IV database is a publicly available dataset containing de-identified patient data established following approval by the Institutional Review Boards of the BIDMC and the Massachusetts Institute of Technology. MIMIC-IV data is sourced from two in-hospital database systems: a custom hospital-wide EHR *HOSP* module and an ICU-specific clinical information system *ICU* module. Data extraction was managed by three of the co-authors (YC, LA and AN) who followed the established guidelines for accessing the MIMIC-IV database. A total of 364,627 unique subjects were identified in MIMIC-IV v3.1, each with a unique subject ID. These subjects had a total of 546,028 hospitalizations with 94,458 ICU stays.

### Defining septic shock

ICU stays that lasted less than 24 h were excluded. In the MIMIC-IV dataset of the 94,458 stays, 54,711 were 24 h or longer. Among these stays, 17,025 (31.1%) met the Sepsis-3 definition [1, 33]. Following this, we determined the onset of septic shock for those 17,025 sepsis stays. Given *t*_*sepsis*_ is the time of sepsis onset, our definition of septic shock was,

1. Stays with vasopressors in the temporal window [*t*_*sepsis*_ – 12 h to ICU discharge].
2. Stays with lactate ≥ 2 mmol/L in the temporal window [*t*_*sepsis*_ – 24 h to ICU discharge].

We defined stays that meet both criteria as septic shock stays, with the onset time (*t*_*septic shock*_) being the time of the first vasopressor initiation. We observed a total of 5,533 stays that met both septic shock criteria (8,781 sepsis stays received vasopressors; 7,643 sepsis stays recorded a lactate ≥ 2 mmol/L). Additional details on the dataset and on the definition of sepsis and septic shock are provided in the supplementary material.

### Defining corticosteroid treatment

Of the 5,533 septic shock stays, 1,009 (18.23%) received one or more doses of a corticosteroid (hydrocortisone, dexamethasone, or methylprednisolone) in the temporal window [_*septic shock*_ – 12 h to ICU discharge] (treatment group), and the remaining 4,524 stays did not receive any (control group).

### Covariates and outcome extraction

For the septic shock cohort, we extracted clinician-specified covariates for both treatment and control groups. The covariates included demographic variables (count = 4), laboratory measurements including hematologic parameters (count = 31), mechanical ventilation settings (count = 2), vasopressor use (count = 1) and illness severity scores (count = 1). We extracted these covariates 24 h prior to the onset of septic shock (*t*_*septic shock*_) for both groups. If multiple measurements were available, we used the most recent. By extracting the covariates prior to septic shock onset (*t*_*septic shock*_), we ensured they were not influenced by vasopressor use.

We extracted key outcomes, including ICU and hospital mortality, ICU and hospital length of stay (LOS), and the need for renal replacement therapy (RRT), from the MIMIC–IV–derived treatment Table [34]. The duration of vasopressor therapy was also extracted from the MIMIC-IV–derived medication table, which recorded time-stamped infusion rates for seven vasoactive drugs (norepinephrine, epinephrine, dopamine, phenylephrine, vasopressin, dobutamine, and milrinone). To facilitate comparison of doses, all vasoactive drug doses were converted to norepinephrine-equivalent doses (NED) as previously described using the MIMIC-IV data [34].

### Treatment effect estimation framework

We applied the Augmented Inverse Probability Weighting (AIPW), a double-robust estimator, to estimate the treatment effect of corticosteroids on patient outcomes [35]. ICU mortality was the primary outcome, and hospital mortality, ICU length of stay (LOS), Hospital LOS, the need for Renal Replacement Therapy (RRT), and the duration of vasopressor administration were the secondary outcomes. We denoted the stay-level design matrix as $\:X\in\:{R}^{N\times\:D}$, where * N* represented the number of stays, * D* the number of covariates, and  the covariate vector for the -th stay (see Table 1 for the covariates). The treatment variable was denoted as $\:T\in\:{\left\{\mathrm{0,1}\right\}}^{N}$, where $\:{T}_{i}=1$ if the * i*-th stay received corticosteroid treatment, and $\:{T}_{i}=0$, otherwise. The outcome variable was denoted by * Y*, which is binary-valued (for mortality and need for RRT) or real-valued (for LOS and vasopressor duration). See Supplementary Material for more information on target trial emulation and the AIPW estimator.

**Table 1:** Summary statistics of extracted covariates and outcomes for the septic shock cohort

	Missing counts	Total	Control group	Treatment group	SMD [*p*-value]
No. stays (*N*)		5,533	4,524	1,009	
**Demographics**					
Age, years, mean (SD)	0	65.86 (14.43)	66.43 (14.29)	63.30 (14.81)	-0.215 [< 0.001]
Male	0	3502 (63.29)	2904 (64.19)	598 (59.27)	0.101 [0.004]
Weight (kg)	637	82.30 [70 ,97]	82.10 [70,97]	82.60 [69, 100]	0.055 [0.726]
**Lab measurements**					
Lymphocytes count (K/uL)	3,476	1.46 [0.80,2.24]	1.56 [0.90,2.33]	0.93 [0.43,1.58]	0.005 [< 0.001]
Neutrophils count (K/uL)	3,477	10.14 [6.70,14.37]	10.08 [6.77,14.19]	10.37 [6.36,15.32]	0.116 [0.479]
Platelet count, (K/uL)	568	161.00 [116.00,223.00]	159.00 [117.00,215.00]	172.00 [108.00,255.00]	0.148 [0.004]
NLR	3,491	6.56 [3.93,12.33]	6.00 [3.83,10.75]	11.04 [5.75,20.68]	0.45 [< 0.001]
PLR	3,492	98.97 [60.39,190.62]	93.14 [58.85,164.01]	179.69 [81.05,389.64]	0.427 [< 0.001]
BUN, (mg/dL)	1,593	24.00 [16.00,41.00]	23.00 [16.00,38.00]	28.00 [17.00,49.00]	0.217 [< 0.001]
Chloride, (mEq/L)	520	106.00 [101.00,109.00]	106.00 [102.00,109.00]	103.00 [98.00,108.00]	-0.386 [< 0.001]
Creatinine, (mg/dL)	1,593	1.20 [0.80,1.90]	1.10 [0.80,1.80]	1.30 [0.80,2.20]	0.173 [< 0.001]
PTT, (Seconds)	974	33.00 [28.50,42.20]	32.65 [28.40,40.90]	35.80 [28.70,48.10]	0.176 [< 0.001]
Lactate, mg/dL	1,231	24.32 [18.92,34.23]	24.32 [18.92,33.33]	24.32 [16.21,38.73]	0.134 [0.436]
**Mech. Vent. settings**					
Mechanical ventilation	0	3587 (64.83)	2980 (65.87)	607 (60.16)	0.119 [0.001]
FiO2	3,909	60 [50, 100]	60 [50, 100]	70 [50, 100]	0.1 [0.153]
**Vasopressor Usage**					
Max vasopressor dose (NED mcg/kg/min)	134	0.07 [0.05,0.14]	0.07 [0.05,0.12]	0.10 [0.05,0.20]	0.108 [< 0.001]
**Scores**					
Max SOFA score	63	5.00 [3.00,8.00]	5.00 [3.00,7.00]	6.00 [4.00,9.00]	0.422 [< 0.001]
Outcomes					
ICU LOS, days	0	4.92 [2.29,11.00]	4.29 [2.17,9.64]	8.75 [3.96,16.92]	0.447 [< 0.001]
Hosp. LOS, days	0	12.00 [6.00,22.00]	11.00 [6.00,20.00]	17.00 [8.00,29.00]	0.345 [< 0.001]
Renal replacement therapy,	0	1104 (19.95)	716 (15.83)	388 (38.45)	0.526 [< 0.001]
Hosp. Mortality	0	1534 (27.72)	1027 (22.70)	507 (50.25)	0.597 [< 0.001]
ICU Mortality	0	1302 (23.53)	859 (18.99)	443 (43.90)	0.557 [< 0.001]
Vasopressor duration, minutes,	0	1908 [587, 4922]	1546 [457, 4173]	3692 [1618, 8423]	0.439 [< 0.001]

Only select labs are shown. For the full list, please see supplementary material. All categorical parameters presented as percentages, Continuous parameters, except for age and duration of administration of vasopressors are presented as median [IQR]

### Model training

We randomly split the * N* stays into five folds. We use the stays in * k-1* folds to train the treatment-effects estimation model. The trained model is then applied to stays in the * k*-th fold to estimate the treatment effects. The model training proceeds in two steps,

1. We trained a propensity score model $\:e\left(x\right)=P(T=1\:|\:X=x)\:$ to characterize the probability of receiving treatment given the covariate vector * X=x*.
2. We trained the treatment outcome prediction model, $$\:{\mu\:}_{1}\left(x\right)$$, and the control outcome prediction model, $$\:{\mu\:}_{0}\left(x\right)$$. For binary-valued outcomes $$\:{\mu\:}_{1}\left(x\right)=P(Y=1\:|\:T=1,\:X=x);$$. $$\:{\mu\:}_{0}(x)=P(Y=1\:|\:T=0,\:X=x)$$ For real-valued outcomes, $$\:{\mu\:}_{1}\left(x\right)=P\left(Y\right|\:T=1,\:X=x);$$$$\:{\mu\:}_{0}(x)=P(Y|\:T=0,\:X=x)$$, where * T* was the treatment variable, * Y* was the outcome variable, and * X* was the covariate vector.

We used the XGBoost classifier for the propensity score model and for the treatment/control outcome prediction models for binary-valued outcomes. We used the XGBoost regressor for the treatment/control outcome prediction models for real-valued outcomes.

We chose the XGBoost model because it can naturally handle missing values in the design matrix, avoiding the need for imputation. When training the XGBoost model, we optimized the hyper-parameters using the standard nested 5-fold cross validation setup.

### Treatment effect estimation

Given the trained models, for the test stays $\:{x}_{j}$, we computed the potential treatment outcome $\:{Y}_{1j}$ and potential control outcome $\:{Y}_{0j}$ as follows,

$$\begin{aligned}\:{Y}_{1j}=&{\mu\:}_{1}\left({X}_{j}\right)+\frac{{T}_{j}}{e\left({X}_{j}\right)}\left({Y}_{j}-{\mu\:}_{1}\left({X}_{j}\right)\right);\\&{Y}_{0j}={\mu\:}_{0}\left({X}_{j}\right)+\frac{1-{T}_{j}}{1-e\left({X}_{j}\right)}\left({Y}_{j}-{\mu\:}_{0}\left({X}_{j}\right)\right).\end{aligned}$$

In this way, we obtained the potential treatment outcomes for all test stays across all 5 folds. To mitigate the effects of train/test splits, we repeated model training and treatment effect estimation * M* times, assigning stays randomly across the 5 folds.

For each stay, we computed the distribution of potential treatment outcomes as $\:p\left({Y}_{1j}\right)$ and the distribution of potential control outcomes as $\:p\left({Y}_{0j}\right)$, where $\:p\left({Y}_{1j}\right)$ and $\:p\left({Y}_{0j}\right)$ were vectors of length * M*. The * j*-th stay was determined to benefit from corticosteroid treatment if the mean of $\:p\left({Y}_{1j}\right)$ was significantly less than the mean of $\:p\left({Y}_{0j}\right)$. We used a two-sample t-test to compare the mean of $\:p\left({Y}_{1j}\right)$ < mean $\:p\left({Y}_{0j}\right)$ at a significance level $\:\alpha\:$. We set the beneficial indicator $\:{B}_{j}=1$ if the * j*-th stay benefits and 0 otherwise.

Lastly, we were interested in identifying covariates indicative of the beneficial indicator $\:{B}_{j}=1$. This translated into identifying haematological parameters indicative of patients likely to benefit from corticosteroid treatment. We computed univariate $\:AUROC\left(F,\:B\right)$ to evaluate the concordance between the covariate * F* and the beneficial indicator * B*. When $\:AUROC\left(F,\:B\right)>0.5$, this was interpreted as higher values of the covariate * F* being associated with a higher likelihood of benefiting from corticosteroid treatment. When $\:AUROC\left(F,\:B\right)<0.5\:$this was interpreted as lower covariate values being associated with a higher likelihood of benefiting from corticosteroid treatment. For each covariate, the greater the deviance from 0.5, the stronger the association with the beneficial indicator. We ranked the covariates by taking the absolute value ($\:\left|AUROC\left(F,\:B\right)-\:0.5\right|$) to accommodate deviation in both directions from 0.5. When computing this metric, we only utilized stays where there were no missing values.

## Results

Table 1 shows the characteristics of the cohort of stays extracted, including demographics and statistics for the extracted covariates and outcomes. Based on these statistics, the treatment group is younger but substantially sicker, with higher SOFA scores, longer ICU stays, higher vasopressor doses, worse renal and metabolic markers (higher BUN/creatinine, lower albumin), and markedly elevated inflammatory indices (NLR, PLR) compared with the control group. The treatment group also experienced longer ICU and hospital LOS and relatively higher rates of mortality and RRT, indicating that corticosteroids were preferentially administered to a sicker subpopulation.

### Evaluation of potential benefit of corticosteroid treatment on ICU mortality

We trained and tested the AIPW estimator using ICU mortality as the primary outcome. We set the number of train/test splits, * M* = 100, and the level of significance for the t-test, $\:\alpha\:$ = 0.05. For the propensity score and treatment outcome XGBoost models, we performed a grid search on the training partitions over two parameters: number of estimators (10, 20, 50, 100, 200, 500, 1000) and learning rate (0.001, 0.01, 0.1, 0.2, 0.3, 0.4, 0.5, 0.6, 0.7, 0.8, 0.9, 1.0 in 0.1 increments). All other settings were set to their defaults.

Among the 5,533 stays with septic shock, the AIPW estimator indicated that corticosteroid use was potentially beneficial in 1,425 stays (25.7%). Table 2 presents the 2 × 2 grid of the treatment and control groups and model-estimated benefit and model-estimated NO benefit groups. We observed that 566 of 1,009 stays (56%) in the treatment group were labelled as potentially benefiting from corticosteroids by the AIPW estimator, while 859 of 4,524 stays (19%) in the control group were likely to benefit from corticosteroid treatment. If we assume that the management of septic shock in the MIMIC-IV dataset reflects modern accepted practice, the 443 stays that received corticosteroids and were deemed unlikely to benefit highlight the need for greater precision in prescribing these medications.

**Table 2:** Comparison of treatment, control groups and model-estimated benefit, model-estimated NO benefit groups for the primary outcome of ICU mortality

	Model-estimated NO benefit group	Model-estimated benefit group	Total
**Treatment ** **group**	443	566	1,009
**Control group**	3,665	859	4,524
**Total**	4,108	1,425	5,533

Table 3 compares the demographic features and clinical covariates of the model-estimated no benefit and benefit groups, respectively. We observed that the patient demographics across the two groups were similar, but disease severity differed markedly. Patients likely to benefit from steroid treatment had a longer median length of stay (7 days), higher maximum SOFA scores (SOFA of 7), and higher vasopressor doses (0.1 mcg/Kg/min) than patients unlikely to benefit.

**Table 3:** Comparison of demographic and clinical covariates between the model-estimated NO benefit and benefit groups for the primary outcome of ICU mortality

Variable	Model-estimated NO benefit group (*N* = 4,108 stays)	Model-estimated benefit group (*N* = 1,425 stays)
**Age (years)**	68 [58, 76] (4,108)	67 [57, 77] (1,425)
**Gender (Female)**	35% (4,108)	41% (1,425)
**Weight (kg)**	82.5 [70, 97] (3,732)	82 [69.2, 97] (1,164)
**Vasopressor dose (mcg/kg/min)**	0.06 [0.05, 0.11] (4,066)	0.1 [0.05, 0.2] (1,333)
**Maximum SOFA score**	5 [3, 7] (4,092)	7 [4, 9] (1,378)
**Length of ICU stay (days)**	4.2 [2.1, 9.8] (4,108)	7.0 [3.6, 14.0] (1,425)

Data presented as [IQR] and (count)

Table 4 shows the top five covariates associated with reduced ICU mortality. For each covariate, the univariate AUROC (i.e. $\:AUROC\left(F,\:B\right)$) and the covariate’s median (IQR) are shown in the model-estimated NO benefit and benefit groups. The count for each covariate used to compute the median (IQR) is also displayed.

The septic shock stays that were more likely to benefit from corticosteroid treatment had higher max SOFA scores, lower lymphocyte counts, higher PLR, NLR and PTT. The univariate AUROC for beneficial indicators of PLR and NLR was primarily driven by variation in lymphocyte count, as the AUROC for platelet and neutrophil counts showed no significant differences between the two groups. The univariate AUROC for the covariates with beneficial indicators was modest (0.60–0.65).

**Table 4:** The top five ranked covariates predicting reduced ICU mortality with corticosteroids

Covariate	AUROC	Model-estimated NO benefit group (*N* = 4,108)	Model-estimated benefit group (*N* = 1,425)
**Maximum SOFA score**	0.65	5 [3, 7] (4,092)	7 [4, 9] (1,378)
**Lymphocyte count (K/uL)**	0.65	1.6 [0.9, 2.3] (1,602)	1.0 [0.5, 1.7] (455)
**PLR**	0.63	91.9 [58.5, 158.5] (1,591)	146.9 [75.5, 327.7] (450)
**NLR**	0.62	5.9 [3.8, 10.7] (1,593)	9.3 [4.9, 19.2] (449)
**PTT (Seconds)**	0.61	32. [28.3, 39.5] (3,487)	37.2 [29.8, 52.0] (1,072)

Data presented as [IQR] and (count)

### Evaluating potential immortal time bias

One common concern associated with the target trial emulation approach is immortal time bias. To evaluate for possible immortal time bias, we repeated our analysis and restricted the treatment group to patients who received corticosteroids within 24 h of shock onset, so that treatment status is anchored to a single time point applied consistently across both groups.

This reduced the number of patient stays in the treatment group from 1,009 (complete cohort) to 629 (patient stays that received steroids within 24 h of shock onset). The median [IQR] of the time between the onset of shock and administration of steroids was 3.46 [0.45, 0.63] hours. The control group remains unchanged. Using this reduced treatment group and control group, we estimated the benefits of both primary and secondary outcomes using the AIPW estimator. All other details are the same. Below we report model-estimated benefits for the primary outcome (Table 5). The results for secondary outcomes on this reduced cohort is included in the Supplementary material.

**Table 5:** The top five ranked covariates predicting reduced ICU mortality with corticosteroids using only cases that received steroids within 24 h of shock onset

Covariate	AUROC	Model-estimated NO benefit group (*N* = 3,943) IQR (count)	Model-estimated benefit group (*N* = 1,210) IQR (count)
**Max SOFA score**	0.67	5 [3, 7] (3,928)	7 [4, 9] (1,166)
**Lymphocyte count**	0.65	1.6 K/uL [1.0, 2.4] (1,524)	1.0 K/uL [0.5, 1.7] (394)
**BUN**	0.63	21.5 mg/dL [15, 34] (2,600)	32 mg/dL [19, 55] (1,020)
**PLR**	0.63	89.7 [58.0, 153.3] (1,531)	138.7 [75.3, 327.5] (389)
**NLR**	0.63	5.8 [3.8, 10.3] (1,533)	9.2 [4.9, 19.2] (388)

Despite some changes in the values presented, the predictive power of the lymphocyte count, NLR and PLR is very similar. Same observations hold for the secondary outcomes; these results are available in the Supplementary material.

### Interaction analysis between haematological parameters

We performed interaction analysis between the different haematological parameters. We examined the effect of corticosteroids between different lymphocyte count groups. We divided all patient stays with available lymphocyte count into four subgroups of equal sample size. The lymphocyte count bin boundaries are 0.80 K/uL (25% percentile), 1.46 K/uL (50% percentile) and 2.24 K/uL (75% percentile), respectively. See Table 6 below,

**Table 6:** Interaction Analysis between lymphocyte count and Model-estimated benefit for ICU mortality

Range of lymphocyte count	Total number of patient stays in the subgroup	Number (%) of patient stays with Model-estimated benefit
≤ 0.80 K/uL	516	188 (36%)
(0.8 K/uL, 1.46 K/uL]	514	121 (24%)
(1.46 K/uL, 2.24 K/uL]	513	79 (15%)
> 2.24 K/uL	514	67 (13%)

As shown in Table 6, the subgroup with the lowest levels of lymphocyte count (≤ 0.8 K/uL), 36% of patients were predicted to benefit from steroid use, significantly higher than the remaining subgroups (confirmed by a chi-square test). As the lymphocyte count increased, the percentage of patients predicted to benefit from steroid use steadily decreased. We performed similar analysis for NLR. For NLR, we observed that the percentage of patients predicted to benefit from steroid use increased as NLR increased (using cutoff values of 3.93, 6.56, and 12.33). The same pattern holds for PLR (using cutoff values of 60.39, 98.97, and 190.63). These results illustrate the relationship between lower lymphocyte count and model-estimated benefit.

### Potential benefit of corticosteroid treatment on secondary outcomes

Similar calculations were performed to estimate the potential benefits of corticosteroids using secondary outcomes, hospital mortality, the need for RRT, ICU LOS, hospital LOS, and vasopressor duration. We performed this analysis on the complete cohort. In Table 7, for each secondary outcome, we show the top five covariates associated with reduced risk. For all secondary outcomes, two covariates consistently ranked among the top five predictors of benefit: the maximum SOFA score and lymphocyte count. The NLR and PLR also ranked highly in four of the five secondary outcomes, suggesting that these covariates have some value in predicting which patients will benefit from corticosteroids. These covariates consistently ranked among the top for both primary and secondary outcomes, with AUROCs ranging from 0.60 to 0.65.

**Table 7:** AUROC of the top five covariates when used to determine model-estimated benefit vs. no benefit for each secondary outcome

Hospital mortality	ICU LOS	Hospital LOS	Need for RRT	Vasopressor duration
Lymphocytes count: 0.66	Lymphocytes count: 0.63	Lymphocytes count: 0.60	Max SOFA score: 0.67	NLR: 0.61
Max SOFA score: 0.66	PLR: 0.63	NLR: 0.60	Creatinine: 0.65	Lymphocytes count: 0.60
NLR: 0.64	NLR: 0.62	PLR: 0.60	Chloride: 0.64	Max SOFA score: 0.60
PLR: 0.63	Max SOFA score: 0.61	Max SOFA score: 0.60	NLR: 0.63	Max vasopressor dosage: 0.60
BUN: 0.61	Max vasopressor dose: 0.59	Chloride: 0.58	Lymphocytes count: 0.62	PLR: 0.58

The covariates are ranked by AUROC. Comparison of AUROC for clinical benefit

Finally, we sought to determine if variation in lymphocyte count independently predicted our primary outcome after adjusting for disease severity as indicated by maximum SOFA score and vasopressor dose. This would indicate whether changes in lymphocyte count were simply a reflection of illness severity or added independent predictive value. We trained a standalone logistic regression model that used three covariates: max SOFA score, max vasopressor dose, and lymphocyte count to predict patient benefit from steroid use for ICU mortality. The learned coefficients are shown in Table 8.

**Table 8:** An evaluation of the interaction between lymphocyte count and severity of illness

	Coefficient of logistic regression
**Constant / bias term**	0.47
**Max SOFA score**	0.27
**Max vasopressor dose**	-0.15
**Lymphocytes count**	-1.13

After adjusting for disease severity, lymphocyte count remained predictive of patient stays likely to benefit from steroid use. This suggests that the variation in lymphocyte count was independently associated with the primary outcome.

## Discussion

This retrospective analysis sought to utilise readily available haematological parameters to identify a subgroup of septic shock cases likely to benefit from corticosteroid administration. We found 5,533 instances of septic shock, of which 1,425 (25.7%) were identified as potentially benefiting from corticosteroids regarding the risk of ICU mortality. For the primary outcome of ICU mortality, lymphocyte count had the greatest predictive ability with an AUC of 0.65. A lower lymphocyte count and associated increases in NLR and PLR were associated with a higher probability of benefiting from corticosteroids across multiple clinically relevant outcomes. This association remained significant even after adjusting for disease severity, suggesting that lymphocyte count was an independent indicator of this specific host response state.

Our results support the prevailing literature indicating that sepsis and septic shock have defined subtypes [13, 14]. Using a variety of inflammatory measures, hypo- and hyperinflammatory types of sepsis have been described in several studies [36, 37]. However, robust evidence linking subtype identification to changes in therapy and improved mortality is still being developed [38, 39]. Advanced measures of immune response and inflammation, such as transcriptomics, proteomics, and assays of various cytokines, are promising approaches but seem impractical as widely used modalities to guide therapy [39, 40]. There is a need for more clinical studies linking the knowledge of these subtypes to changes in therapy and improved mortality [38, 39].

Regarding corticosteroids in septic shock, determining which patients will benefit is only one essential question to answer. The ideal timing in the disease process to start corticosteroids remains largely unanswered [41]. A quick review of specific studies suggests faster reversal of shock but no effect on other outcomes like mortality or the need for renal replacement therapy [42, 43]. Regarding the ideal dose of corticosteroids, the Surviving Sepsis guidelines recommend 200 mg of hydrocortisone daily [44]. It is clear that high doses (> 400 mg/day) are associated with increased complications without increased benefit [45]. However, it is unclear if doses lower than 200 mg/day are associated with different outcomes [46]. The matter is further complicated by the results of the APROCCHSS study, which found that adding 50 ug of fludrocortisone to 200 mg of hydrocortisone daily results in decreased mortality [12].

The lymphopenia that defines our “Model-estimated benefit” group suggests sepsis-related immune paralysis, affecting the adaptive immune response [47]. There were a few examples of the use of inflammatory markers to predict the response to corticosteroids in septic shock. Comparisons with the available similar studies were very mixed. Wong et al. defined two subtypes of pediatric patients with septic shock using 100 genes that influence immune function and glucocorticoid expression [48]. Patients in the subtype with more complete suppression of adaptive immunity were more likely to do worse on corticosteroids than those with a more varied suppression. This result seems somewhat different from our results, in which patients with more suppressed acquired immunity benefit from corticosteroids. Our data were consistent with the results reported by Bologheanu, indicating that leucocytes play a central role in predicting benefit, but no details were provided on the effects of increasing or decreasing values [32]. The study by Rajendran evaluated patients with sepsis, from a variety of causes, and the results were similar in that reduced adaptive immunity was associated with benefit from steroids [30]. Our results differed from those of Wittermans et al., who examined patients with pneumonia and found that increased NLR and PLR predicted benefit from steroids, but this was primarily due to increased neutrophil counts [31]. Sun and colleagues evaluated the combination of lymphocyte counts and inflammatory biomarkers in predicting benefit with corticosteroids in septic patients [24]. Patients with low lymphocyte counts and low inflammatory markers were more likely to benefit from corticosteroids than other patients.

This raises questions of the mechanisms by which corticosteroids produce a therapeutic effect. It highlights gaps in our understanding of the pathophysiology of sepsis and septic shock, how they evolve over the clinical course, and how they vary across different sites of infection, such as pneumonia. Correction of sepsis-induced adrenal insufficiency as the primary mechanism is not well supported by the literature, and current recommendations do not support evaluating adrenal function to predict benefit from corticosteroid therapy [49, 50]. A cursory classification of corticosteroids as immunosuppressants makes their role counterintuitive in the setting of immune paralysis. The mechanism by which steroids confer benefit is likely more complex than simply suppressing the host immune response and inflammation. They are better described as immunomodulators [51, 52], with their effects on immune function varying by infection location, patient characteristics and timing and dose of steroids. A careful review of the literature shows that corticosteroids are not always immunosuppressive. A Cochrane review on the use of steroids in pneumonia found that corticosteroids did not increase the risk of other infections [53]. The HYPOLYTE study found reduced risk of secondary pneumonia in trauma patients who received hydrocortisone [54]. Another large study of surgical patients found reduced risk of sepsis in patients treated with dexamethasone [55]. One large Cochrane meta-analysis found no increased risk of superinfection in patients with sepsis who received corticosteroids [56].

The concept of monitoring a rapidly changing immune response was eloquently described in the work of Monneret almost 20 years ago [57]. This initial work described an early inflammatory phase followed by a later phase of immune paralysis and highlighted the need to individualize therapy based on the phase of immune dysfunction [57]. Members of this research group have more recently suggested that the NLR may be a low-cost way of immune monitoring in sepsis, comparing it to the less available mHLA-DR, which they suggest is the reference for evaluating immune dysfunction in sepsis [29, 58]. While this work has focused on the use of lymphocyte count, NLR and PLR to guide corticosteroids, it may be a cheap and widely available biomarker to guide many of the new immunomodulatory therapies [59].

Lymphocyte count and NLR have AUROC values for predicting model-estimated benefit for ICU mortality between 0.62 and 0.65, which can only be described as modest discriminatory power. This is not unexpected in a complex, multifactorial syndrome like septic shock. There is currently no gold standard for predicting the benefit of corticosteroid therapy with which to compare these results. Guidelines recommend their use in patients with more severe illness, as indicated by a poor response to fluid resuscitation and vasopressors, or by the SOFA score [44, 60]. The SOFA score had similar predictive power to lymphocyte count, NLR, and PLR in this study. Hellali et al. evaluated the predictive power of several machine learning models in 1241 patients from the APROCCHSS study and found the highest AUC of 0.72 with logistic regression [61]. A quick review of the literature suggests the modest values of 0.62 to 0.65 should not be dismissed.

Our approach does offer a significant pragmatic advantage. Unlike measurements of specific inflammatory cytokines or evaluation of transcriptomic signatures, which are not widely available, the NLR, PLR and lymphocyte count are derived from the complete blood count, an inexpensive test that is part of the daily routine in virtually every ICU worldwide [62]. This inherent simplicity and accessibility make our findings potentially translatable and scalable, particularly in resource-limited settings where more sophisticated and costly biomarkers are not feasible. Despite the compelling science around measuring inflammatory cytokines, genomic or transcriptomic signatures, there is little real-world data yet, and their use is limited to a few academic centres.

In our study, the absolute lymphocyte counts outperformed both NLR and PLR for 3 of the 5 stated outcomes. Neutrophil and platelet counts were not predictive of response to corticosteroids, and clinically significant changes in the NLR and PLR were driven largely by variation in the lymphocyte count. This raises the question of the added value of the NLR and PLR over the lymphocyte count in predicting response to corticosteroids. We believe that at this stage, it is best to evaluate all parameters in future research in this area. We note possible differences between the relationship between these parameters and corticosteroids in sepsis and septic shock. Considering sepsis and septic shock as distinct points in the same dynamic process, our results represent a snapshot of this process at a single point in time. Consideration of the trajectories of lymphocyte count and the NLR has shown promise in prognostication in sepsis and may provide added value [63, 64].

This study has several limitations. Its retrospective nature precludes definitive causal inference. Although the use of a robust double-robust estimator (AIPW) minimises confounding, residual or unmeasured confounders may still exist. The researchers also note missing data for key parameters. This remains a concern despite the ability of the XGBoost learning algorithm to deal with missing data. The analysis is based on data from a single, large tertiary care centre (BIDMC), and the findings require external validation. The absence of site of infection as a variable is also a concern. There is significant evidence that patients with lung infections may benefit from steroid therapy [17, 65]. Finally, the timing, dose, and type of corticosteroid were not standardized, reflecting real-world practice but introducing potential variability in the response.

It is premature to recommend any changes to clinical practice based on this single retrospective study; however, our findings suggest potential avenues for future research. If validated prospectively, these simple biomarkers could offer a step towards a more nuanced approach to corticosteroid use in septic shock. Rather than a definitive decision-making tool, they might serve as a signal, prompting clinicians to consider a patient’s underlying immune status. This could help identify patients for whom the risk-benefit ratio of corticosteroid therapy is more favourable, thereby guiding enrollment in future clinical trials.

Future research should focus on prospectively validating these signals. Correlation with more advanced markers of inflammation and immune response will also be valuable. An endotype-guided randomized controlled trial, in which patients with septic shock are stratified by baseline lymphocyte count, NLR or PLR before randomization, would be the definitive next step to determine whether this biomarker-guided strategy can truly improve outcomes. Evaluating changes in these values over time may provide additional information and is a simple, straightforward step toward understanding this clinical question. If future research supports the utility of these markers in this setting, they may find a role as a practical way to monitor sepsis-related immune function as part of a more complex scoring system and as a simple, low-cost tool to assist in guiding therapy in septic shock.

## Conclusion

In patient stays with septic shock, we observed an association between lower lymphocyte counts (and higher NLR) and potential benefit from corticosteroid therapy. The predictive power of these markers is modest but consistent; they are simple, widely available, and perform comparably to more complex severity scores in identifying potential benefit. These hypothesis-generating findings warrant prospective validation to determine if this biomarker-guided approach can refine the use of corticosteroids in this critically ill population.

## Supplementary Information

Below is the link to the electronic supplementary material.

Supplementary Material 1

## Data Availability

The MIMIC-IV data is freely available; credentialed access can be gained by following the steps outlined below. https://physionet.org/content/mimiciv/
