# Supplementary material for: Lymphopenia and elevated NLR identify patients with septic shock who may benefit from corticosteroids: a target trial emulation study
Source: Intensive Care Med Exp. 2026 Sep 29;14:127. doi: 10.1186/s40635-026-00972-8 (PMC13624219; doi:10.1186/s40635-026-00972-8)
Supplement: Supplementary file 1 [file 40635_2026_972_MOESM1_ESM.docx]

**Supplementary Material**

**Dataset**

The Medical Information Mart for Intensive Care (MIMIC-IV) database is a comprehensive collection of deidentified medical records from patients admitted to the Beth Israel Deaconess Medical Centre's emergency department and intensive care unit in Boston, MA. The database, encompassing data from over 65,000 ICU patients and 200,000 emergency department visits, aims to enhance patient care through knowledge discovery and algorithm development [1]. MIMIC-IV builds on the success of its predecessor, MIMIC-III, and introduces a modular approach to data organization, facilitating easier integration and use of various data sources.

MIMIC-IV is sourced from two in-hospital database systems: a custom hospital-wide EHR *HOSP* module and an ICU-specific clinical information system *ICU* module. A total of 364,627 unique subjects are in MIMIC-IV v3.1, each represented by a unique subject ID. These subjects had 546,028 hospitalizations and 94,458 unique ICU stays.

**Sepsis Onset**

In this research, we used the *ICU* module to extract the subjects with septic shock. To determine onset of septic shock, we first determined the onset of sepsis. Subjects that spent less than 24 hours in the ICU were excluded. The clinical onset of sepsis was defined according to the Sepsis-3 guidelines.[2], which we briefly describe here,

1. *t_suspicion_*: The time of clinical suspicion is either the time of antibiotics followed by blood cultures within 24 hours or the time of blood cultures followed by antibiotics within 72 hours. In both cases, the antibiotics should have been administered continuously for at least 72 hours.
2. *t_SOFA_*: The time when the sequential organ failure assessment (SOFA) score dropped by 2 or more units within a 24-hour period.
3. *t_sepsis_*: The time of clinical onset of sepsis. Note that *t_SOFA_* will need to occur within 48 hours before *t_suspicion_* or 24 hours after *t_suspicion_* (i.e., *t_suspicion_* - 48 ≤ *t_SOFA_* ≤ *t_suspicion_* + 24). When this criterion is satisfied, the clinical onset time of sepsis is the minimum of [*t_SOFA_*, *t_suspicion_*].

We assumed that the baseline SOFA score was 0 before ICU admission in patients not known to have pre-existing organ dysfunction. The creators of the MIMIC-IV dataset have previously computed and provided a derived table with sepsis onset times using Sepsis-3 guidelines [2, 3].

**Extracted demographics, covariates and outcomes**

**Table S1: Full list of summary statistics of extracted covariates and outcomes for the septic shock cohort**

|  | Missing Counts | Both  Groups | Control  Group | Treatment Group | SMD  [p-value] |
| --- | --- | --- | --- | --- | --- |
| No. stays (N) |  | 5,533 | 4,524 | 1,009 |  |
| **Demographics** |  |  |  |  |  |
| Age (years)  mean (SD) | 0 | 65.86  (14.43) | 66.43 (14.29) | 63.30  (14.81) | -0.215 [<0.001] |
| Male sex (n (%)) | 0 | 3502  (63.29) | 2904  (64.19) | 598  (59.27) | 0.101 [0.004] |
| Weight (kg) | 637 | 82.30  [70 ,97] | 82.10  [70,97] | 82.60  [69, 100] | 0.055 [0.726] |
| **Lab measurements** |  |  |  |  |  |
| Base Excess, (mEq/L) | 1,429 | -1  [-4, 0] | -1.00  [-4, 0] | -2  [-6, 0] | -0.14 [<0.001] |
| Bicarbonate (mEq/L) | 1,736 | 23  [20, 26] | 23  [20, 26] | 22  [19, 26] | -0.041 [0.078] |
| pH | 1,428 | 7.35  [7.29,7.40] | 7.35  [7.30,7.40] | 7.33  [7.26,7.40] | -0.207 [<0.001] |
| Partial pressure CO_2_ (mmHg) | 1,429 | 42.00 [37.00,48.00] | 42.00 [37.00,48.00] | 43.00 [36.00,52.00] | 0.185 [0.096] |
| Oxygen saturation, (%) | 4,508 | 97.00 [93.00,98.00] | 97.00 [94.00,98.00] | 96.00 [90.00,98.00] | -0.202 [<0.001] |
| Aspartate Aminotransferase (IU/L) | 3,476 | 56.00 [29.00,135.00] | 56.00 [29.00,142.00] | 57.00 [29.00,127.25] | 0.003 [0.928] |
| BUN (mg/dL) | 1,593 | 24.00 [16.00,41.00] | 23.00 [16.00,38.00] | 28.00 [17.00,49.00] | 0.217 [<0.001] |
| Alkaline phosphatase (IU/L) | 3,489 | 90.00 [63.00,143.00] | 86.00 [62.00,132.00] | 102.00 [69.00,164.75] | 0.167 [<0.001] |
| Calcium (mg/dL) | 2,379 | 8.40 [7.80,9.00] | 8.40 [7.80,9.00] | 8.40 [7.80,9.00] | 0.016 [0.874] |
| Chloride (mEq/L) | 520 | 106.00 [101.00,109.00] | 106.00 [102.00,109.00] | 103.00 [98.00,108.00] | -0.386 [<0.001] |
| Creatinine (mg/dL) | 1,593 | 1.20 [0.80,1.90] | 1.10 [0.80,1.80] | 1.30 [0.80,2.20] | 0.173 [<0.001] |
| Bilirubin direct (mg/dL) | 5,356 | 1.80 [0.70,5.40] | 1.70 [0.60,5.65] | 2.10 [0.83,4.17] | -0.132 [0.691] |
| Magnesium (mg/dL) | 2,266 | 2.10 [1.90,2.30] | 2.10 [1.90,2.30] | 2.10 [1.90,2.30] | 0.024 [0.306] |
| Phosphate (mg/dL) | 2,398 | 3.90 [3.10,5.00] | 3.90 [3.10,5.00] | 4.00 [3.10,5.30] | 0.119 [0.088] |
| TroponinT (ng/mL) | 4,615 | 0.18 [0.05,0.73] | 0.20 [0.06,0.88] | 0.11 [0.04,0.40] | -0.19 [0.001] |
| Albumin (g/dL) | 4,193 | 3.10 [2.50,3.70] | 3.10 [2.50,3.70] | 2.90 [2.40,3.50] | -0.232 [<0.001] |
| Platelet (K/uL) | 568 | 161.00 [116.00,223.00] | 159.00 [117.00,215.00] | 172.00 [108.00,255.00] | 0.148 [0.004] |
| PTT (seconds) | 974 | 33.00 [28.50,42.20] | 32.65 [28.40,40.90] | 35.80 [28.70,48.10] | 0.176 [<0.001] |
| Lactate (mg/dL) | 1,231 | 24.32 [18.92,34.23] | 24.32 [18.92,33.33] | 24.32 [16.21,38.73] | 0.134 [0.436] |
| Potassium (mEq/L) | 2,275 | 4.20 [3.80,4.60] | 4.20 [3.80,4.60] | 4.20 [3.70,4.70] | 0.118  [0.58] |
| Fibrinogen (mg/dL) | 2,698 | 199.00 [157.00,264.00] | 199.00 [159.00,259.00] | 197.00 [141.00,325.75] | 0.21  [0.976] |
| WBC (K/uL) | 635 | 12.80 [9.10,17.50] | 12.80 [9.20,17.40] | 12.40 [8.40,18.12] | 0.056 [0.226] |
| Bilirubin total (mg/dL) | 3,501 | 0.90 [0.50,2.40] | 0.90 [0.50,2.20] | 1.00 [0.50,3.10] | 0.114 [0.122] |
| Glucose (mg/dL) | 487 | 150.00 [123.00,184.75] | 151.00 [124.00,183.00] | 146.00 [114.00,196.75] | 0.064 [0.346] |
| Hemoglobin, g/dL | 426 | 9.70 [8.40,11.20] | 9.70 [8.40,11.10] | 9.80 [8.30,11.40] | 0.053 [0.406] |
| Hematocrit, % | 422 | 29.40 [25.85,33.90] | 29.20 [25.80,33.50] | 30.00 [26.00,35.30] | 0.129 [0.003] |
| Lymphocytes count K/uL | 3,476 | 1.46 [0.80,2.24] | 1.56 [0.90,2.33] | 0.93 [0.43,1.58] | 0.005 [<0.001] |
| Neutrophils count K/uL | 3,477 | 10.14 [6.70,14.37] | 10.08 [6.77,14.19] | 10.37 [6.36,15.32] | 0.116 [0.479] |
| CRP, mg/L | 5,456 | 102.00 [74.40,170.20] | 101.00 [78.25,167.05] | 111.80 [68.25,174.07] | 0.114 [0.517] |
| NL-Ratio | 3,491 | 6.56 [3.93,12.33] | 6.00 [3.83,10.75] | 11.04 [5.75,20.68] | 0.45 [<0.001] |
| PL-Ratio | 3,492 | 98.97 [60.39,190.62] | 93.14 [58.85,164.01] | 179.69 [81.05,389.64] | 0.427 [<0.001] |
| **Mech. Vent. settings** |  |  |  |  |  |
| Mechanical ventilation (n (%)) | 0 | 3587 (64.83) | 2980 (65.87) | 607 (60.16) | 0.119 [0.001] |
| FiO2 (%) | 3,909 | 60  [50, 100] | 60  [50, 100] | 70  [50, 100] | 0.1  [0.153] |
| **Vasopressor Usage** |  |  |  |  |  |
| Max vasopressor dose | 134 | 0.07 [0.05,0.14] | 0.07 [0.05,0.12] | 0.10 [0.05,0.20] | 0.108 [<0.001] |
| **Scores** |  |  |  |  |  |
| Maximum SOFA score, | 63 | 5.00 [3.00,8.00] | 5.00 [3.00,7.00] | 6.00 [4.00,9.00] | 0.422 [<0.001] |
| **Outcomes** |  |  |  |  |  |
| ICU LOS (days) | 0 | 4.92 [2.29,11.00] | 4.29 [2.17,9.64] | 8.75 [3.96,16.92] | 0.447 [<0.001] |
| Hospital LOS (days) | 0 | 12.00 [6.00,22.00] | 11.00 [6.00,20.00] | 17.00 [8.00,29.00] | 0.345 [<0.001] |
| Received renal replacement therapy (n (%)) | 0 | 1104 (19.95) | 716 (15.83) | 388 (38.45) | 0.526 [<0.001] |
| Hospital Mortality (n (%)) | 0 | 1534 (27.72) | 1027 (22.70) | 507 (50.25) | 0.597 [<0.001] |
| ICU Mortality (n (%)) | 0 | 1302 (23.53) | 859 (18.99) | 443 (43.90) | 0.557 [<0.001] |
| Duration of vasopressor therapy (minutes) | 0 | 1908 [587, 4922] | 1546 [457, 4173] | 3692 [1618, 8423] | 0.439 [<0.001] |
| **(mcg/kg/min)** | | | | | |

**Target Trial Emulation with AIPW Estimator**

Target trial emulation is a causal inference framework for estimating treatment effects from retrospective observational data when a randomised controlled trial (RCT) has not been conducted. The gold standard for determining the benefit of corticosteroids in septic shock would be an RCT. A target trial emulation uses observational data to recreate the design of an RCT, as closely as possible. We use this approach not to establish definitive causation, but to generate hypothesis-generating estimates that can guide the design of future prospective studies.

Looking at the primary outcome of ICU mortality within this framework, each patient stay has two possible treatments (steroids, no steroids) and two potential outcomes under each (survival, death). Only one of these outcomes is ever observed for a given patient, under the treatment they received, while the outcome they would have had under the alternative treatment remains counterfactual. This is the central challenge of causal inference from observational data: we never see what would have happened to the same patient under both treatment strategies.

One of the problems with trying to answer a clinical question like this one with observational data is that the risk of the outcome (death) is not equal in the two treatment groups at baseline. Our data clearly indicate that sicker patients were more likely to receive steroids. The AIPW estimator addresses this by estimating both potential outcomes for each patient: P(Y1j), the estimated probability of mortality had patient j received steroids, and P(Y0j), the estimated probability of mortality had they not, even though only one of these was observed. Comparing these estimated probabilities lets us approximate the treatment effect for each patient without requiring random assignment. This allows us to ask what would have happened under the alternative treatment, using data in which that alternative was never actually tried.

**Results**

**Table S2: The top five ranked covariates predicting reduced hospital mortality with corticosteroids on complete cohort**

| **Covariate** | **AUROC** | **Cohort with no Model-estimated benefit (N=4,004)** | **Cohort with Model-estimated benefit (N=1,529)** |
| --- | --- | --- | --- |
| **Lymphocyte count (K/uL)** | 0.66 | 1.6  [0.9, 2.3] (1,583) | 1.0  [0.5, 1.7] (474) |
| **Maximum SOFA score** | 0.66 | 5  [3, 7] (3,988) | 7  [4, 9] (1,482) |
| **NLR** | 0.64 | 5.9  [3.8, 10.4] (1,574) | 10.1  [5.0, 20.1] (468) |
| **PLR** | 0.63 | 90.7  [58.2, 157.0] (1,572) | 153.3  [77.7, 327.7] (469) |
| **BUN (mg/dL)** | 0.61 | 22.0  [15.0, 35.0] (2,661) | 30.0  [19.0, 52.0] (1,279) |
| **Presented as [IQR] and (count).** | | | |

**Table S3: The top five ranked covariates associated with reduced ICU Length of stay with corticosteroids on complete cohort**

| **Covariate** | **AUROC** | **Cohort with no Model-estimated benefit (N=3,691)** | **Cohort with Model-estimated benefit (N=1,842)** |
| --- | --- | --- | --- |
| **Lymphocyte count (K/uL)** | 0.63 | 1.6  [1.0, 2.4] (1,455) | 1.1  [0.6, 1.8] (602) |
| **PLR** | 0.63 | 87.9  [57.3, 151.0] (1,447) | 138.6  [75.4, 290.1] (594) |
| **NLR** | 0.62 | 5.7  [3.7, 10.4] (1,448) | 9.0  [5.1, 17.3] (594) |
| **Maximum SOFA score** | 0.61 | 5  [3, 7] (3,654) | 6  [4, 9] (1,816) |
| **Max Vasopressor dose (mcg/kg/min)** | 0.59 | 0.06  [0.05, 0.11] (3,623) | 0.10  [0.05, 0.20] (1,776) |
| **Presented as [IQR] and (count).** | | | |

**Table S4: The top five ranked covariates most associated with reduced hospital length of stay with corticosteroids on complete cohort**

| **Covariate** | **AUROC** | **Cohort with no Model-estimated benefit (N=3,503)** | **Cohort with Model-estimated benefit (N=2,030)** |
| --- | --- | --- | --- |
| **Lymphocyte count (K/uL)** | 0.60 | 1.6  [1.0, 2.3] (1,344) | 1.2  [0.6, 2.0] (713) |
| **NLR** | 0.60 | 5.8  [3.7, 10.3] (1,377) | 8.5  [4.6, 16.6] (705) |
| **PLR** | 0.60 | 89.2  [57.8, 154.2] (1,336) | 126.3  [69.1, 263.5] (705) |
| **Maximum SOFA score** | 0.60 | 5  [3, 7] (3,456) | 6  [4, 9] (2,014) |
| **Chloride (mEq/L)** | 0.58 | 106  [102, 109] (3,240) | 105  [100, 109] (1,773) |
| **Presented as [IQR] and (count).** | | | |

**Table S5: The top five ranked covariates most associated with reduced risk of needing renal replacement therapy (RRT) with corticosteroids on complete cohort**

| **Covariate** | **AUROC** | **Cohort with no Model-estimated benefit (N=4,196)** | **Cohort with Model-estimated benefit (N=1,337)** |
| --- | --- | --- | --- |
| **Maximum SOFA score** | 0.67 | 5  [3, 7] (4,165) | 7  [4, 10] (1,305) |
| **Creatinine (mg/dL)** | 0.65 | 1.1  [0.8, 1.6] (2,821) | 1.6  [1.0, 3.1] (1,119) |
| **Chloride (mEq/L)** | 0.64 | 106  [103, 110] (3,840) | 103  [98, 108] (1,173) |
| **NLR** | 0.63 | 5.9  [3.7, 10.8] (1,613) | 9.6  [5.4, 17.6] (429) |
| **Lymphocyte count**  **(K/uL)** | 0.62 | 1.6  [0.9, 2.3] (1,623) | 1.1  [0.6, 1.8] (434) |
| **Presented as [IQR] and (count).** | | | |

**Table S6: The top five ranked covariates most associated with reduced vasopressor duration with corticosteroids on complete cohort**

| **Covariate** | **AUROC** | **Cohort with no Model-estimated benefit**  **(N=3,672)** | **Cohort with Model-estimated benefit**  **(N=1,861)** |
| --- | --- | --- | --- |
| **NLR** | 0.61 | 5.9  [3.7, 10.5] (1,421) | 8.6 [4.6, 16.8] (621) |
| **Lymphocyte count (K/uL)** | 0.60 | 1.6  [0.9, 2.3] (1,430) | 1.2  [0.6, 2.0] (627) |
| **Maximum SOFA score** | 0.60 | 5  [3, 7] (3,645) | 6  [4, 9] (1,825) |
| **Maximum Vasopressor dose (mcg/kg/min)** | 0.60 | 0.06  [0.05, 0.10] (3,632) | 0.10  [0.05, 0.20] (1,767) |
| **PLR** | 0.58 | 92.6  [58.6, 164.8] (1,420) | 121.8  [67.5, 264.0] (621) |
| **Presented as [IQR] and (count).** | | | |

**Evaluating Potential Immortal Time Bias**

One common concern associated with this approach is immortal time bias. Unlike an RCT where patients would randomly receive steroids or no steroids from the start of the study, patients may start steroids later during their septic shock and be considered in the steroid group. As a result, patients must survive long enough after the onset of shock to receive corticosteroids, so defining "steroid treatment" based on exposure at any point during a stay will erroneously credit survival to the steroids. To address this, we repeated our analysis and restricted the treatment group to patients who received corticosteroids within 24 hours of shock onset, excluding those who started treatment later, so that treatment status is anchored to a single time point applied consistently across both groups.

This reduced the number of patient stays in the treatment group from 1009 (complete cohort) to 629 (patient stays that received steroids within 24 hours). The median [IQR] of the time between the onset of shock and administration of steroids was 3.46 [0.45, 0.63] hours. The control group was unchanged. Using this reduced treatment group and control group, we estimated the benefits of both primary and secondary outcomes using the AIPW estimator. All other details are the same. The model-estimated benefits for both primary and secondary outcomes are presented below.

**Table S7: The top five ranked covariates predicting reduced ICU mortality with corticosteroids using only cases that received steroids within 24 hours of shock onset (reduced cohort)**

| **Covariate** | **AUROC** | **Model-estimated NO benefit Group (N=3,943)**  **IQR (count)** | **Model-estimated benefit Group (N=1,210)**  **IQR (count)** |
| --- | --- | --- | --- |
| **Max SOFA score** | 0.67 | 5  [3, 7] (3,928) | 7  [4, 9] (1,166) |
| **Lymphocyte count** | 0.65 | 1.6 K/uL  [1.0, 2.4] (1,524) | 1.0 K/uL  [0.5, 1.7] (394) |
| **BUN** | 0.63 | 21.5 mg/dL  [15, 34] (2,600) | 32 mg/dL  [19, 55] (1,020) |
| **PLR** | 0.63 | 89.7  [58.0, 153.3] (1,531) | 138.7  [75.3, 327.5] (389) |
| **NLR** | 0.63 | 5.8  [3.8, 10.3] (1,533) | 9.2  [4.9, 19.2] (388) |

**Table S8: AUROC of the top five covariates when used to determine model-estimated benefit vs. no benefit for each secondary outcome. The covariates are ranked by AUROC on reduced cohort**

| **Hospital mortality** | **ICU LOS** | **Hospital LOS** | **RRT** | **Vasopressor duration** |
| --- | --- | --- | --- | --- |
| Max SOFA score: 0.67 | Max SOFA score: 0.62 | Max SOFA score: 0.60 | Creatinine: 0.70 | Max SOFA score: 0.62 |
| Lymphocyte count: 0.66 | Lymphocyte count: 0.61 | NLR: 0.59 | Max SOFA score: 0.70 | NLR: 0.61 |
| NLR: 0.64 | NLR: 0.60 | Lymphocyte count: 0.58 | Chloride: 0.65 | Max SOFA score: 0.60 |
| PLR: 0.64 | PLR: 0.59 | PLR: 0.58 | BUN: 0.65 | Lymphocyte count: 0.60 |
| BUN: 0.64 | Max vasopressor dose: 0.58 | Albumin: 0.58 | NLR: 0.63 | Chloride: 0.58 |

The discriminability power of the maximum SOFA scores improved slightly from 0.65 to 0.67. However, there was very limited change in the overall results, and the lymphocyte count and other haematological parameters remained predictive of several of the study outcomes. This suggests that immortal time bias does not play a significant role in these findings.

**Evaluating the relative contribution of neutrophil, lymphocyte, and platelet counts to the predictive power of NLR and PLR**

Further, we investigated the predictive ability of lymphocyte count when used in NLR and PLR. We trained four standalone logistic regression models as,

1. Model A: used neutrophil count to predict the benefit indicator
2. Model B: used both neutrophil count and NLR to predict the benefit indicator
3. Model C: used lymphocyte count to predict the benefit indicator
4. Model D: used both lymphocyte count and NLR to predict the benefit indicator

For each model, we conducted 5-fold cross-validation to obtain five AUROC scores on the test folds. A t-test was applied to compare the performance differences between the different models. The mean and standard deviation of AUROC scores of each model are shown in Table S9 below.

**Table S9: Comparing the predictive power of neutrophil and lymphocyte count in relation to NLR for ICU Mortality**

| **Model: inputs** | **Mean (std) AUC from 5-fold cross-validation** |
| --- | --- |
| A: neutrophil count | 0.53 (0.01) |
| B: neutrophil count and NLR | 0.65 (0.01) |
| C: lymphocyte count | 0.65 (0.02) |
| D: lymphocyte count and NLR | 0.62 (0.02) |

First, using neutrophil count achieved a mean AUROC of 0.53 (model A), which is only slightly better than the random chance of 0.50. This implied neutrophil count has extremely limited predictive power. After adding NLR as the input (model B), the mean AUROC increased to 0.65. A t-test confirms that the increase is statistically significant (p-value < 1e-4). This indicates that NLR has much more predictive power than neutrophil count.

Second, using lymphocyte count achieved a mean AUROC of 0.65 (model C), which is equal to that achieved by combining neutrophil count and NLR (model B). Furthermore, after combining NLR and lymphocyte count as inputs (model D), the mean AUROC slightly decreased to 0.62. A t-test confirms the performance difference between model D and model C is not statistically significant (p-value = 0.1). This means NLR does not contain additional predictive power when compared to lymphocyte count.

The comparison between model A and model B, and the comparison between model C and model D, show the predictive ability of NLR is largely driven by lymphocyte count. We also reached similar conclusions for PLR and lymphocyte count.

**Supplementary Material References**

1.	Aew J, L B, L S, et al (2023) MIMIC-IV, a freely accessible electronic health record dataset. Sci Data 10:. https://doi.org/10.1038/s41597-022-01899-x

2.	Singer M, Deutschman CS, Seymour CW, et al (2016) The Third International Consensus Definitions for Sepsis and Septic Shock (Sepsis-3). JAMA 315:801–810. https://doi.org/10.1001/jama.2016.0287

3.	Aew J, J A, Jd R, et al (2018) A Comparative Analysis of Sepsis Identification Methods in an Electronic Database. Crit Care Med 46:. https://doi.org/10.1097/CCM.0000000000002965
